# Analysis of non-prospective trial registration in clinical trials submitted to *The BMJ*: observational study

**DOI:** 10.1136/bmj-2025-086467

**Published:** 2026-02-18

**Authors:** David Blanco, Elizabeth Loder, Sophie Cook, Martí Casals, Jordi Cortés, Aïda Cadellans-Arróniz, Victor Zárate, Ella Hubbard, Sara Schroter

**Affiliations:** 1 Department of Physiotherapy, Universitat Internacional de Catalunya, 08195 Sant Cugat del Vallès, Barcelona, Spain; 2 Department of Neurology, Brigham and Women’s Hospital, Boston, MA, USA; 3 *The BMJ*, London, UK; 4 National Institute of Physical Education of Catalonia (INEFC), University of Barcelona, Barcelona, Spain; 5 University of Vic-Central University of Catalonia (UVic-UCC), Barcelona, Spain; 6 Research group in Biostatistics and Bioinformatics (GRBIO), Department of Statistics and Operations Research, and Institute for Research and Innovation in Health (IRIS), Universitat Politècnica de Catalunya, Barcelona, Spain; 7 Faculty of Public Health and Policy, London School of Hygiene and Tropical Medicine, London, UK

## Abstract

**Objectives:**

To identify variables associated with non-prospective registration of clinical trials submitted to *The BMJ*; examine deficiencies in registration, disclosure of deficiencies, and subsequent publication status of such trials; and assess the authors’ claims of prospective registration.

**Design:**

Observational study.

**Setting:**

*The BMJ,* London.

**Population:**

239 of 287 submissions to *The BMJ* (2019-23) reporting clinical trial results as defined by the International Committee of Medical Journal Editors (ICMJE) and flagged by editors as potentially not prospectively registered, and 239 trials prospectively registered in an ICJME accepted registry from a randomised list of all clinical trial submissions in the same period.

**Main outcome measures:**

Study outcomes included non-prospective registration in an ICJME accepted registry and, for non-prospectively registered trials, deficiencies in registration deficiencies in registration (retrospective in an ICJME accepted registry, registration in a non-ICJME accepted registry, or no registration), delay in registration of retrospectively registered trials, and subsequent publication status. Time to publication and disclosure of registration deficiencies on publication were also assessed, along with the journal’s impact factor and whether the journal claimed adherence to the ICMJE registration recommendations.

**Results:**

Reduced odds of non-prospective registration in an ICMJE accepted registry were associated with larger sample sizes (101-500 *v* 1-100; odds ratio 0.43, 95% confidence interval 0.22 to 0.84), corresponding authors from Oceania (reference: Europe; 0.35, 0.14 to 0.82), a greater number of authors (10 *v* 5; 0.71, 0.59 to 0.87), mention of Consolidated Standards Of Reporting Trials (0.22, 0.06 to 0.67), more recent submissions (2021-23 *v* 2019-20; 0.63, 0.42 to 0.95), and presence of funding (eg, non-profit *v* no funding or no information; 0.20, 0.09 to 0.41). Higher odds were observed for corresponding authors based in Asia (reference: Europe; 1.75, 1.07 to 2.89). Of 176 trials not prospectively registered in an ICMJE accepted registry submitted between 2019 and 2021, 146 (83%) were retrospectively registered (median delay 193 days), 23 (13%) were unregistered, and seven (4%) were registered in a non-ICJME accepted registry. Most (155 trials, 88%) were subsequently published; 138 (89%) of these appeared in journals with an impact factor (median 5.39) and 96 (62%) in journals claiming adherence to the ICMJE recommendations. The median time from initial submission to *The BMJ* to publication was 12 months. Only about one sixth explicitly disclosed registration deficiencies on publication. Of 72 authors responding about prospective registration on submission, 60 (83%) incorrectly claimed adherence.

**Conclusions:**

Many trials rejected by *The BMJ* for non-prospective registration in an ICMJE accepted registry were later published in high impact journals claiming adherence to the ICMJE recommendations, often without disclosure of registration deficiencies.

## Introduction

Prospective clinical trial registration promotes transparency and is important for preventing selective publication and selective reporting of research outcomes, reducing duplication of research efforts, keeping patients and the public informed about active or planned trials, and providing ethics committees with relevant background information on similar studies.^1^ Prospective trial registration is also associated with a lower risk of bias.^2^ In 2004, the International Committee of Medical Journal Editors (ICMJE) first mandated that to be eligible for publication in ICMJE member journals, all clinical trials that started enrolment after 1 July 2005 should be registered prospectively (that is, on or before the enrolment date of the first participant) in a trial registry meeting several criteria.^3^ In June 2007, the ICMJE started accepting registration in any of the primary registries of the World Health Organization’s (WHO) International Clinical Trials Registry Platform (ICTRP), as well as in ClinicalTrials.gov, the Dutch Trial Register, EudraCT, and the UMIN Clinical Trials Registry.^1^ A systematic review showed that rates of prospective trial registration increased from 3% in 2009 to 21% in 2013.^4^ Although more recent systematic reviews are lacking, individual studies analysing trials registered or published up to 2022 have reported improved yet still suboptimal rates: 58.6% in rheumatology^5^ and 69% in trials from Switzerland.^6^ Previous studies have identified several factors associated with prospective trial registration, including region, sample size, multicentre recruitment, and industry funding.^4^ ^5^ ^6^ ^7^ However, these variables, alongside others such as intervention type, trial design, and mention of CONSORT (Consolidated Standards Of Reporting Trials) have not been systematically examined across submissions to high impact, general medical journals with a global scope, such as *The BMJ*. Examining these variables could inform more effective trial registration policies and help direct resources or educational efforts towards research contexts, such as certain regions, intervention types, or designs, with lower adherence to registration requirements.

Despite increased attention to trial registration, most previous research on registration practices has focused on published trials rather than on submission of manuscripts. One study analysing published clinical trials from 2010 to 2015 found that high impact medical journals claiming adherence to the ICMJE recommendations often published retrospectively registered trials.^8^ Even among ICMJE member journals, only around two thirds of trials were prospectively registered, although this proportion was higher than in non-ICMJE member journals.^7^ Only one study examined submissions of manuscripts describing trials.^9^ Focusing on submissions rather than on published trials is important because it allows registration practices to be assessed before editors or peer reviewers have had an input, providing a clearer picture of adherence to ICMJE requirements at the point of manuscript submission. Such focus also provides an opportunity to examine what happens to trials that were not prospectively registered in an ICMJE accepted registry. This study found that 97% of trials submitted to *The BMJ* between 2013 and 2017 were not prospectively registered in an ICMJE accepted registry and were subsequently published in journals with a median impact factor of 4.97. Only 2.9% of these trials disclosed problems with registration upon publication. Although these studies provide invaluable insights, two decades after the introduction of the ICMJE mandate, up-to-date evidence is still lacking on trial registration practices across submissions to high impact, general medical journals. Specifically, it remains unclear how often trials not prospectively registered are published in journals claiming adherence to the ICMJE recommendations, how long it takes to publication, the types of registration deficiencies commonly observed, and the extent to which authors disclose non-prospective registration on submission and upon publication. Addressing these gaps is critical for understanding ongoing challenges to adherence and for informing strategies to enhance the transparency of trial registration.

We identified variables associated with non-prospective registration in an ICMJE accepted registry among clinical trial reports submitted to *The BMJ* between 2019 and 2023; examined the types of registration deficiencies, as well as subsequent publication status and disclosure of these deficiencies in publications of trials not prospectively registered in an ICMJE accepted registry when submitted to *The BMJ*; and assessed the proportion of retrospectively registered trials where the authors claimed their trial was prospectively registered on submission to *The BMJ.*

## Methods

### Study design and setting

This observational study analysed clinical trial reports submitted to *The BMJ* between 2019 and 2023. Submissions were accessed under a research agreement with BMJ Group. During this period, 287 trials were flagged by *The BMJ*’s editors as appearing to be non-prospectively registered in an ICMJE accepted registry. Following our independent review, we confirmed and included 239 of these trials as not being prospectively registered in an ICMJE accepted registry. To identify variables associated with lack of prospective registration, we additionally selected a random sample of 239 clinical trial submissions from the same period that we verified had been prospectively registered in an ICMJE accepted registry. To do this, we exported a list of all research manuscripts submitted to *The BMJ* during this period and identified those with any of the following keywords in the title or abstract: “trial”, “randomised”, “randomized”, “controlled”, or “RCT”. We then randomised the order of all these submissions, and a single evaluator verified each in turn to confirm that it was a clinical trial and that it had been prospectively registered in an ICMJE accepted registry. This process was continued until we reached 239 verified trials.

We collected and analysed data between December 2024 and May 2025. To produce this report and ensure transparent and complete reporting, we followed STROBE (Strengthening the Reporting of Observational Studies in Epidemiology) guidelines.^10^

### Eligibility criteria

Research manuscripts were eligible if they reported clinical trial results and had been submitted to *The BMJ* between 1 January 2019 and 31 December 2023. We used the ICMJE definition of a clinical trial: “any research project that prospectively assigns people or a group of people to an intervention, with or without concurrent comparison or control groups, to study the relationship between a health-related intervention and a health outcome.”^11^ Clinical trial protocols and observational analyses of clinical trials were excluded, but we included secondary analyses of trials. We distinguished observational from secondary analyses by reviewing the title and abstract. Observational analyses were categorised as those that used trial data in an observational manner without experimental assignment, and secondary analyses involved additional analyses (eg, long term follow-up) that retained the trial’s experimental design.

### Variables and data sources

We categorised all trials as prospectively registered or not in an ICMJE accepted registry on submission to *The BMJ*. Prospective registration in an ICMJE accepted registry was defined as registration in any registry accepted by the ICMJE on or before the enrolment date of the first participant.^1^ The ICMJE accepts 22 unique registries,^1^ including the 18 primary registries recognised by the WHO ICTRP,^12^ which meet specific criteria for content, quality and validity, accessibility, unique identification, technical capacity, and administration, plus ClinicalTrials.gov, the Dutch Trial Register, EudraCT, and the UMIN Clinical Trials Registry (see supplementary information file 1 for full list of ICMJE accepted registries). As recommended by the ICMJE, we used the date on which trial registration materials were first submitted to a registry as the date of registration.^11^ Rather than relying on the trial registry, we used the enrolment date of the first participant as reported in the submitted manuscript or associated submission documentation. This approach was taken because others have observed that authors of retrospectively registered trials sometimes alter the trial start date in the registry to a time point after registration, creating the false impression that the trial was prospectively registered.^13^

Trials that were not prospectively registered in an ICMJE accepted registry were classified as having registration deficiencies, defined as one of the following: retrospective registration in an ICJME accepted registry—the trial was registered in a registry accepted by the ICMJE after the date the first participant was enrolled according to the submitted manuscript; registration in a non-ICJME accepted registry—the trial was registered, either prospectively or retrospectively, in a registry not accepted by the ICMJE; and no registration—the trial was not registered in any trial registry.

#### Identifying variables associated with non-prospective registration in an ICMJE accepted registry

We captured several variables associated with non-prospective registration in an ICMJE accepted registry: study characteristics, author and institutional attributes, specific mention of using or adhering to CONSORT, year of submission, and funding source.

*Study characteristics*—trial design (parallel group, cluster, crossover, adaptive, factorial, or non-randomised), trial setting (multicentre or single centre trial), sample size (number of participants assigned to study interventions: 1-100, 101-500, or ≥501 participants), and type of intervention (drug or non-drug). Supplementary information file 2 provides further details on the classification of drug interventions.

*Author and institutional attributes*—number of authors and corresponding author’s institutional region. Regions were grouped as Africa and the Middle East, Asia, Oceania, Central and South America, Europe, or North America following a previously used categorisation.^9^ Africa and the Middle East and Central and South America were combined owing to the low volume of submissions, whereas Oceania was reported separately because of its substantial volume of submissions.

*Specific mention of CONSORT*—claimed use or adherence to CONSORT guidelines^14^ or any of its extensions^15^ beyond the inclusion of a CONSORT checklist.

*Year of submission*—manuscripts submitted between 2019 and 2023.

*Funding source*—non-profit organisations (including universities, national government, local government, research institutes and centres, libraries and data archiving organisations, or other non-profit organisations); associations and societies, and trusts, charities, or foundations (both private and public); international organisations; for profit companies (including the pharmaceutical industry); no funding (where no funding entity was identified); or no information (where no information related to funding was provided). We followed a previously used categorisation.^16^

These variables were extracted from multiple sources: submission year and corresponding author’s institutional region were obtained from *The BMJ*’s manuscript system; the number of authors and mention of CONSORT were automatically categorised from the manuscript full texts using text matching techniques implemented in Python.^17^ We validated the automated categorisation on a random sample of 48 manuscripts (10% of the total). For detecting CONSORT mentions, sensitivity was 100%, specificity was 97.1%, and overall accuracy was 97.9% compared with human evaluation; for number of authors, accuracy was 87.5%. In the six articles with discrepancies in the number of authors, the extracted counts all deviated by only one author, indicating minimal errors.

A single evaluator manually categorised all other variables (intervention type, sample size, trial design, trial setting, and funding source). For the last three of those variables, we automatically extracted information from the relevant manuscript subheadings to facilitate manual categorisation. We did not consult trial registries for these data. The code used for text matching extracted data is available on Zenodo.^18^

#### Registration deficiencies, publication status, and subsequent disclosure of deficiencies

We assessed several variables of trials not prospectively registered in an ICMJE accepted registry submitted to *The BMJ* between 2019 and 2021. To minimise the risk of misclassification of more recent submissions as unpublished, we excluded the most recent trials (submitted in 2022 and 2023) from this analysis.

*Registration deficiencies*—defined in the section entitled “Variables and data sources.”

*Registration delay*—for retrospectively registered trials, we calculated the delay (in days) between the enrolment date of the first participant as supplied on submission of the manuscript and the trial registration date in the trial registry.

*Publication status*—whether the trial was published as a full length article in a journal after initial submission to *The BMJ* (preprints, abstracts, research letters, and working papers were excluded), including manuscripts that were submitted to and rejected by *The BMJ* but later submitted to and published by other journals. We determined publication status primarily through Google searches using the article title and trial registration number, when available. If this was unsuccessful, we tried author searches on PubMed and ORCID. For published trials:

• publication in a journal with or without an impact factor. If the journal had a journal impact factor in the year of publication, we extracted it from Clarivate’s Journal Citation Reports;

• publication in a journal claiming adherence to the ICMJE recommendations according to the ICMJE website (before April 2025 when this list ceased to exist)^19^; and time to publication in a journal (in months);

• disclosure of deficiencies in registration upon publication—that is, no explicit mention of non-prospective registration in an ICMJE accepted registry or trial registration and enrolment dates; no explicit mention of non-prospective registration, but mention of both dates; or explicit mention of non-prospective registration; and

• for published trials that were unregistered on submission to *The BMJ*, whether they were retrospectively registered after this submission date.

#### Assessing the validity of authors’ claims of prospective registration

We examined responses to the submission question “Is it [your trial] prospectively registered?” provided by authors of retrospectively registered trials on submission to *The BMJ*. This analysis was intended to assess the consistency between authors’ self-reported and actual registration practices. The analysis was not restricted to trials not prospectively registered in an ICMJE accepted registry, as for other study objectives, because the original question did not specify which registries *The BMJ* accepted.

### Statistical analysis

For our descriptive analysis, we reported frequencies and percentages for all categorical variables, both overall and stratified by whether the trial was or was not prospectively registered in an ICMJE accepted registry. Our dataset had no missing data.

We used two approaches to assess the association between the study variables and non-prospective registration in an ICMJE accepted registry. Firstly, we fitted univariable logistic regression models, with non-prospective registration in an ICMJE accepted registry as the dependent variable and each study variable independently included as the explanatory variable. We estimated odds ratios with 95% confidence intervals (CIs) and corresponding P values for each model coefficient. Global P values were reported using likelihood ratio tests for categorical variables with more than two levels. Secondly, we conducted a multivariable analysis by fitting a logistic regression model with non-prospective registration in an ICMJE accepted registry as the dependent variable and all study variables as explanatory variables. The initial model included all candidate variables, which had been chosen based on previous evidence and theoretical relevance when available. A backward stepwise selection process was applied, whereby variables were sequentially removed. At each step, we used the bayesian information criterion combined with likelihood ratio tests to identify the variable most suitable for removal. A variable was removed if neither of the two criteria provided evidence that it was relevant. Additionally, before and after each removal, we qualitatively assessed that changes to the coefficients of the remaining variables were not excessively large, to ensure that no important confounder was inadvertently excluded.^20^ This process was repeated until no further variables could be removed without an important loss of model performance. We tested all possible two way interactions between the categorical variables in the final multivariable model to explore whether the associations between study variables and non-prospective registration in an ICMJE accepted registry differed across levels of other variables included in the model. Only those interactions were kept that statistically significantly improved model fit based on likelihood ratio tests. For all coefficients in the final model, we reported adjusted odds ratios with 95% CIs. Model validation was performed through residual analysis and calibration.

Before fitting the models, we conducted an exploratory analysis to determine the appropriate categorisation of categorical variables and to assess whether continuous variables required transformation. For each variable, we evaluated several approaches, including linear, quadratic, log transformed, and categorical specifications. The most appropriate option was determined by comparing model fit statistics (bayesian information criterion and Akaike information criterion) and inspecting residuals. Based on this analysis, we grouped categories for three categorical variables in both the univariable and the multivariable analyses to improve model performance: for trial design, we combined the three categories with fewer cases (crossover, factorial, and adaptive trials); for region, we combined both Africa and Middle East and Central and South America for the same reason; and for funding, we grouped no funding with no information, and associations and societies with international organisations. In addition, we categorised two of the three numerical variables to improve model fit: year of submission—a highly discrete variable—into two groups (2019-20 and 2021-23) and sample size into three categories (1-100, 101-500, and ≥501). For the sample size categorisation, we followed a previous study with the addition of the ≥501 category to account for the large number of trials with high sample sizes.^8^ The variable number of authors was retained as a continuous variable but was modelled using a quadratic term. The R code, which is available on Zenodo, provides further details and rationale for these decisions.^21^

For the analysis of registration deficiencies, publication status, and subsequent disclosure of registration deficiencies, we calculated counts and percentages for categorical variables, means and standard deviations (SDs) for continuous variables, and medians with interquartile range (IQR) and deciles (eg, first 10th) for discrete variables.

For the analysis of the validity of authors’ claims of prospective registration, we calculated the count and percentage of authors who incorrectly claimed prospective registration.

All analyses were performed using version 4.5.0 of the R statistical software.^22^ Supplementary information file 3 describes the R packages used. The data and reproducible code used in this study are publicly available.^21^

### Patient and public involvement

No patients or members of the public were involved in the design, conduct, reporting, or dissemination plans of our research. This was a descriptive analysis of documentary evidence. We were not seeking lived experience from patients or the public.

## Results

### Characteristics of included trials

Table 1 presents the characteristics of the 478 included clinical trials, along with a breakdown by prospective versus non-prospective registration in an ICMJE accepted registry. Most trials used a parallel design (344, 72%), were multicentred (277, 58%), and enrolled more than 100 participants (384, 80%). Non-drug interventions were the most common (325, 68%). The majority of corresponding authors were from Europe (239, 50%) and Asia (134, 28%). Most trials had at least 11 authors (283, 60%), and about one third (162, 34%) mentioned CONSORT guidelines. Submissions peaked in 2020 (137, 29%) and more than half of overall submitted trials were funded by non-profit sources (271, 57%).

**Table 1:** Characteristics of the included trials according to registration in an ICMJE accepted registry

Characteristics	All (n=478)	Prospectively registered in ICMJE accepted registry	
		No (%) registered (n=239)	No (%) not registered (n=239)
**Trial characteristics**			
Design:			
Parallel	344 (72)	181 (76)	163 (68)
Cluster	90 (19)	41 (17)	49 (20)
Crossover	15 (3)	8 (3)	7 (3)
Adaptive	5 (1)	2 (1)	3 (1)
Factorial	5 (1)	3 (1)	2 (1)
Non-randomised	19 (4)	4 (2)	15 (6)
Multicentre:			
Yes	277 (58)	146 (61)	131 (55)
No	201 (42)	93 (39)	108 (45)
Sample size:			
1-100	94 (20)	43 (18)	51 (21)
101-500	185 (39)	108 (45)	77 (32)
≥501	199 (42)	88 (37)	111 (46)
Type of intervention:			
Drug	153 (32)	83 (35)	70 (29)
Non-drug	325 (68)	156 (65)	169 (71)
**Author and institutional attributes**			
Corresponding author’s region:			
Africa and Middle East	8 (2)	4 (2)	4 (2)
Asia	134 (28)	54 (23)	80 (33)
Oceania	30 (6)	22 (9)	8 (3)
Central and South America	9 (2)	5 (2)	4 (2)
Europe	239 (50)	125 (52)	114 (48)
North America	58 (12)	29 (12)	29 (12)
No of authors:			
1-5	40 (8)	9 (4)	31 (13)
6-10	152 (32)	67 (28)	85 (36)
11-20	197 (41)	109 (46)	88 (37)
≥21	86 (18)	53 (22)	33 (14)
**Use of reporting guidelines**			
CONSORT mentioned:			
Yes	162 (34)	93 (39)	69 (29)
No	316 (66)	146 (61)	170 (71)
**Year of submission**			
2019	99 (21)	46 (19)	53 (22)
2020	137 (29)	61 (26)	76 (32)
2021	104 (22)	57 (24)	47 (20)
2022	64 (13)	34 (14)	30 (13)
2023	74 (15)	41 (17)	33 (14)
**Funding source**			
Non-profit	271 (57)	152 (64)	119 (50)
Associations and societies	102 (21)	53 (22)	49 (20)
For profit	40 (8)	19 (8)	21 (9)
International organisations	9 (2)	4 (2)	5 (2)
No funding	30 (6)	7 (3)	23 (10)
No information	26 (5)	4 (2)	22 (9)

CONSORT=Consolidated Standards Of Reporting Trials; ICMJE=International Committee of Medical Journal Editors.

### Univariable and multivariable analyses

**Fig 1:**
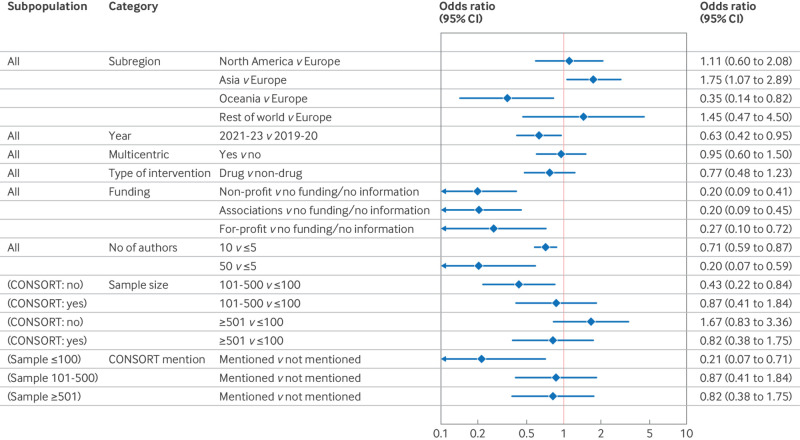
Forest plot of variables associated with non-prospective registration in an ICMJE accepted registry (multivariable model). CI=confidence interval; CONSORT=Consolidated Standards Of Reporting Trials; ICMJE=International Committee of Medical Journal Editors

In the multivariable analysis (table 2), several variables were associated with reduced odds of non-prospective registration in an ICMJE accepted registry, after adjusting for all other variables in the model: larger sample sizes (101-500 participants *v* 1-100; odds ratio 0.43, 95% CI 0.22 to 0.84), a corresponding author from Oceania (reference: Europe; 0.35, 0.14 to 0.82), a greater number of authors (10 authors *v* 5; 0.71, 0.59 to 0.87), CONSORT mentioned (0.22, 0.06 to 0.67), more recent submission year (2021-23 *v* 2019-20; 0.63, 0.42 to 0.95), and any type of funding source (eg, non-profit *v* no funding or no information; 0.20, 0.09 to 0.41; see other categories in table 2). In contrast, trials with corresponding authors based in Asia were associated with increased odds of non-prospective registration in an ICMJE accepted registry compared with those based in Europe (1.75, 1.07 to 2.89). Other variables, such as type of intervention and whether the trial was multicentred, did not show meaningful associations with non-prospective registration in an ICMJE accepted registry. Figure 1 presents the odds ratios and 95% confidence intervals for all variables.

**Table 2:** Univariable and multivariable analyses of variables associated with non-prospective registration in an ICMJE accepted registry

	Univariable analysis			Multivariable analysis	
	Odds ratio (95% CI)	P value		Odds ratio (95% CI)	P value
Trial design (ref: parallel):		0.04			
Cluster	1.33 (0.83 to 2.12)	0.23			
Crossover, adaptive, and factorial	1.03 (0.45 to 2.32)	0.95		—*	—*
Non-randomised	4.16 (1.48 to 14.83)	0.01			
Multicentre (ref: no):		0.16			0.83
Yes	0.77 (0.54 to 1.11)			0.95 (0.60 to 1.50)	
Sample size (ref: 1-100):		0.01			<0.001
101-500	0.60 (0.36 to 0.99)	0.05		0.43 (0.22 to 0.84)	0.01
≥501	1.06 (0.65 to 1.74)	0.81		1.67 (0.83 to 3.37)	0.15
Type of intervention (ref: non-drug):		0.20			0.27
Drug	0.78 (0.53 to 1.14)			0.77 (0.48 to 1.23)	
Region (ref: Europe):		0.01			0.01
North America	1.10 (0.62 to 1.95)	0.75		1.11 (0.60 to 2.08)	0.74
Asia	1.62 (1.06 to 2.50)	0.03		1.75 (1.07 to 2.89)	0.03
Oceania	0.40 (0.16 to 0.9)	0.03		0.35 (0.14 to 0.82)	0.02
Africa and Middle East, and Central and South America	0.97 (0.35 to 2.63)	0.96		1.46 (0.47 to 4.50)	0.51
No of authors (ref: 5 authors):		<0.001			<0.01
10	0.71 (0.60 to 0.84)	<0.001		0.71 (0.59 to 0.87)	0.004
50	0.29 (0.11 to 0.75)	0.01		0.20 (0.07 to 0.59)	0.02
CONSORT mentioned (ref: no):		0.02			<0.001
Yes	0.64 (0.43 to 0.93)			0.22 (0.06 to 0.67)	
Year (ref: 2019-20):		0.04			0.03
2021-23	0.69 (0.48 to 0.99)			0.63 (0.42 to 0.95)	
Funding source (ref: no funding and no information):		<0.001			<0.001
Non-profit	0.19 (0.09 to 0.37)	<0.001		0.20 (0.09 to 0.41)	<0.001
Associations, societies, and international organisations	0.23 (0.10 to 0.48)	<0.001		0.20 (0.09 to 0.45)	<0.001
For-profit	0.27 (0.11 to 0.66)	<0.001		0.27(0.10 to 0.70)	<0.01
[Multivariable model interactions] (ref: sample size 1-100):					
CONSORT mentioned and sample size 101-500				9.32 (2.50 to 39.15)	<0.001
CONSORT mentioned and sample size ≥501				2.27 (0.61 to 9.35)	0.24

CONSORT=Consolidated Standards Of Reporting Trials; ICMJE=International Committee of Medical Journal Editors.

* Removed from final model.

Notably, the final model included an interaction term between mention of CONSORT and sample size. Overall, the probability of non-prospective registration in an ICMJE accepted registry was lower when CONSORT was mentioned. Among studies that did not mention CONSORT, sample size showed no clear association with non-prospective registration in an ICMJE accepted registry. In contrast, among studies that did mention CONSORT, smaller sample sizes (≤100 participants) were associated with significantly lower odds of non-prospective registration in an ICMJE accepted registry (0.22, 0.05 to 0.91).

Model validation was performed using a calibration plot (see supplementary information file 4).

The findings from the univariable analysis were broadly consistent with those of the multivariable model (table 2). Notably, in that analysis, non-randomised trials were associated with significantly increased odds of non-prospective registration in an ICMJE accepted registry compared with parallel group trials (4.16, 1.48 to 14.83).

### Registration, disclosure of deficiencies, and outcome of publication

Most of the 176 trials not prospectively registered in an ICMJE accepted registry submitted to *The BMJ* between 2019 and 2021 were classified as such because of retrospective registration in these registries (146, 83%). A smaller proportion were not registered at all (23, 13%), whereas only a few were registered in a non-ICJME accepted registry (7, 4%). Retrospectively registered trials showed a median delay of 193 days (IQR 64-538 days); first decile=19.4)) between the enrolment date of the first participant reported in the submitted manuscript and the trial registry’s registration date.

Most of the 176 trials not prospectively registered in an ICMJE accepted registry on submission to *The BMJ* were eventually published in a journal (155 trials, 88%), including one in *The BMJ*. Among these 155 trials, 138 (89%) were published in journals with an impact factor (median 5.39 (IQR 3.98-10.40)), and nearly two thirds (96 trials, 62%) were published in journals claiming adherence to the ICMJE recommendations. The median time to publication was 12 months, with 25% of articles published within eight months and 75% within 19 months.

Of the 155 published trials, only 24 (15%) explicitly acknowledged the non-prospective registration in an ICMJE accepted registry. An additional 10 trials (6%) reported both the registration date and the date of first participant enrolment but did not explicitly state that the registration was not prospective in an ICJME accepted registry. The remaining 121 trials (78%) neither acknowledged the non-prospective registration in an ICMJE accepted registry nor reported both the enrolment and the registration dates.

Of the 25 trials that were unregistered at the time of the original submission, nine had not been published in full by 29 April 2025. Of the 14 that were published, about one third (5, 36%) were retrospectively registered after the original submission.

### Authors’ claims of prospective registration

Of the 72 out of 110 trials retrospectively registered on submission to *The BMJ* that responded to the journal’soptional submission question in 2021-23, “Is it [your trial] prospectively registered,” most authors (60; 83%) incorrectly claimed that it was.

## Discussion

This study identified several variables associated with reduced odds of non-prospective registration in an ICMJE accepted registry, including larger sample sizes, corresponding authors affiliated with Oceania, a greater number of authors, mention of CONSORT guidelines, more recent submission years, and the presence of any declared funding source. Retrospective registration in an ICJME accepted registry was more common than registration in a non-ICJME accepted registry or no registration. Despite this, most authors of trials not prospectively registered in an ICJME accepted registry claimed prospective registration on submission. Most of these trials, after rejection by *The BMJ*, were subsequently published in journals with an impact factor, about two thirds of which claimed adherence to the ICMJE recommendations. Notably, only about one in six of the published trials not prospectively registered in an ICMJE accepted registry on submission to *The BMJ* explicitly acknowledged non-prospective registration in the published article.

### Comparison with other studies

Several studies have examined associations between specific trial characteristics and prospective trial registration. Our findings align with previous research, which identified larger sample sizes, a greater number of authors, and region as important predictors of prospective registration.^5^ ^7^ Unlike other studies, however, we did not observe an association with multicentred trials.^5^ ^6^ For type of intervention, the literature presents mixed results: while some studies reported differences in registration rates based on intervention type,^5^ another study focusing on the Australian New Zealand Clinical Trials Registry (ANZCTR)^23^ found no such variation, which is consistent with our findings. In terms of funding, trials with any declared source—whether from non-profit national organisations, professional societies, international agencies, or for profit entities—were statistically significantly more likely to be prospectively registered in an ICMJE accepted registry than those with no funding or unreported funding information. However, prospective registration in an ICMJE accepted registry did not differ statistically significantly across the different funding source types, contrasting with previous studies that observed an association between industry funding and prospective registration.^4^ ^5^ ^8^ Notably, only 40 trials (8%) in our sample were funded by for profit entities, which may have limited our ability to assess this association. Importantly, although we followed the ICMJE definition of prospective registration, some of the other studies on trial registration we have cited used less stringent criteria, allowing delays of up to 30 days and still classifying the trial as prospectively registered.^5^ These different definitions may affect study comparability.

A clear temporal trend was also observed, with more recent trials more likely to be prospectively registered in an ICMJE accepted registry, reflecting ongoing improvements in trial transparency practices over the past decade.^2^ ^4^ ^5^ ^6^ ^8^ These increases may be attributed to the ICMJE mandate and the 2007 US Food and Drug Administration Amendments Act (FDAAA), which requires registration within 21 days of the first participant, as well as to the growing emphasis on transparency in clinical research to prevent selective reporting and publication bias, and the enforcement by clinical trial units and research ethical review boards.^2^ ^4^ ^7^ ^24^ ^25^ Notably, trials in which authors explicitly referenced the CONSORT statement in the manuscript text were more likely to be registered appropriately. This is reassuring as both CONSORT 2010^14^ and CONSORT 2025^26^ include a dedicated item on trial registration. Additionally, the CONSORT for Abstracts checklist recommends including the trial registration number within the structured abstract.^14^ ^26^ Finally, although specific trial designs (eg, parallel group versus crossover) were not statistically significantly associated with non-prospective registration in an ICMJE accepted registry, non-randomised trials were notably less likely to be prospectively registered in an ICMJE accepted registry. We hypothesise that this may be because some investigators conducting non-randomised studies do not perceive their work as qualifying as a clinical trial under the ICMJE definition.

As observed in previous studies, retrospective registration in an ICJME accepted registry was more common than registration in a non-ICJME accepted registry or no registration.^4^ ^5^ ^6^ ^9^ ^23^ The median delay for retrospectively registered trials was 193 days (>6 months), with three quarters delayed by more than 64 days and one quarter delayed by 538 days (almost 18 months), showing that these delays are often substantial and not just a matter of days. In contrast, 95% of retrospectively registered trials in one study^7^ were registered within three weeks of enrolment, whereas in our study, only one in 10 retrospectively registered trials had a delay of less than 19.4 days. One such example was the trial published in *The BMJ* where the authors explained that a registration attempt—made before the enrolment of the first participant—was not recorded owing to an incorrect email address. As a result, the official registration occurred only a few days after the first participant was enrolled. The journal accepted this explanation. However, we argue that late registration should not be overlooked except under very specific and verifiable circumstances, which should be explained in the publication. Delayed registration may reflect poor organisation on the part of investigators, raising concerns about the overall reliability and rigour of the trial. Supporting this concern, one study^27^ showed that even seemingly minor issues, such as simple mathematical errors or small inconsistencies, were often associated with more serious underlying problems upon closer examination.

Most trials not prospectively registered in an ICMJE accepted registry on submission to *The BMJ* (88%) were subsequently published in journals. These journals had a median impact factor of 5.39, with 25% having a journal impact factor greater than 10.40. Our results differ slightly from those of a previous publication,^9^ which found that a higher proportion of non-prospectively registered trials (95.7%) up to 2015 were published in journals with lower impact factors (median 4.97; 25% >6.64). Our new findings challenge even more strongly the assumption that these trials are confined to lower impact or questionable journals. At the same time, we acknowledge that impact factor thresholds vary considerably across specialties, and many high quality trials are published in journals with journal impact factors below this level. Although recent research suggests that journals claiming adherence to the ICMJE recommendations tend to perform better in terms of registration practices,^5^ our findings indicate that most trials not prospectively registered in an ICMJE accepted registry on submission to *The BMJ* were subsequently published in these journals. Because the ICMJE currently has no mechanism to assess adherence to its recommendations, it is particularly important for journals to strengthen their editorial processes to better identify and reject trials not prospectively registered in an ICMJE accepted registry. Among the published trials, only 15% explicitly disclosed non-prospective registration in an ICJME accepted registry or provide basic details, such as enrolment and registration dates, that would allow readers to identify the problem. Although this represents an improvement compared with the 2.9% reported previously for trials up to 2015,^9^ it still indicates that reporting of trial registration remains inadequate. This finding reinforces the need for journals to require the inclusion of key information in submitted manuscripts and in submission systems, such as the exact enrolment and registration dates, the trial registry name and number, and a hyperlink to the registry entry.

Most authors of retrospectively registered trials incorrectly claimed prospective registration on initial submission to *The BMJ*. Possible reasons include researchers believing their study did not meet the ICMJE definition of a clinical trial,^9^ ^28^ being unaware of the trial registration requirements,^9^ ^23^ assuming that obtaining ethical approval was equivalent to trial registration,^9^ ^29^ lacking time and resources,^6^ ^23^ or registering shortly after enrolment, as allowed under certain regulatory frameworks (eg, 2007 US FDAAA).^7^ Indeed, one study reported that many retrospectively registered trials, particularly those based in the US, were registered within three weeks, which is consistent with the 21 day window permitted under the FDAAA.^7^ It is also possible that authors may have misunderstood the question or thought that if they did not indicate prospective registration their paper would be rejected. For all these reasons, we recommend that *The BMJ* revises the wording of the trial registration questions on its manuscript submission system to include a clearer definition of prospective registration and a list of ICMJE accepted registries to better guide authors regarding journal expectations.

### Strengths and limitations of this study

Strengths of this study include the use of data from a high impact general medical journal that receives submissions across diverse clinical specialties, intervention types, and geographical regions, enhancing the relevance and generalisability of the dataset. We manually verified whether each trial was prospectively registered in an ICMJE accepted registry. This step ensured consistency because editorial flags were sometimes inconsistent owing to differences in how editors interpreted registration dates and the often low quality or incomplete reporting of trial registration information in the manuscripts. By standardising the date of first submission in line with ICMJE recommendations, we minimised misclassification and improved the reliability of our registration data. Additionally, we validated the automated categorisation and our statistical models to ensure analytical robustness.

Limitations include the focus on submissions to *The BMJ*, a single high impact journal, which may limit generalisability to journals with different editorial policies, scope, or prestige. For example, our sample showed a skew towards trials with a sample size >100 (80.3%) compared with a previous study, in which 57.6% of trials exceeded this threshold.^8^ This difference may reflect *The BMJ’s* submissions profile, as the journal tends to attract larger trials, even at the submission stage, irrespective of eventual acceptance. Additionally, including the covid-19 pandemic years may have influenced researchers’ behaviour, and editors in some journals may have been more lenient for research they believed needed to be organised, conducted, and published quickly. The optional question “Is it [your trial] prospectively registered?” was only introduced in 2021, limiting sample size and potentially biasing findings on author misreporting. Importantly, our analysis assessed only whether trials mentioned the CONSORT guidelines, not whether they adhered to them. To determine whether a journal claimed adherence to the ICMJE trial registration policy, we used a list provided by the ICMJE before April 2025 (when the list ceased owing to concerns about accuracy).^19^ Additionally, a single evaluator verified the prospectively registered trials and the categorisation of manually coded variables. Finally, we did not preregister this study or make the study protocol publicly available before data collection.

### Implications

Trial registration is a crucial safeguard against misreporting and non-reporting of trial findings. The ICMJE requirement for prospective registration has had a major impact on trial registration behaviour, as the threat of non-publication in influential journals carries important reputational and career consequences for researchers. However, failure to enforce the consequences of non-prospective registration in an ICMJE accepted registry—namely, ineligibility for publication in journals adhering to ICMJE recommendations—dilutes this penalty and reduces incentives to comply with this key aspect of research integrity. Our findings highlight ongoing challenges in ensuring adherence and transparency in trial registration, emphasising the urgent need for stronger enforcement and greater awareness within the research community.

Journals must ensure their policies on trial registration are clearly available on their websites. They could also require that submitted and published manuscripts include both at the end of the abstract and in the main body of the manuscript the date (day/month/year) the trial was first submitted to a registry, the date the submission was approved by the registry (where available), the date the first participant was enrolled in the trial, the name of the registry, the registration number, a hyperlink to the registry entry, and a clear indication whether registration was prospectively registered in an ICMJE accepted registry (box 1). Inclusion of these details both in submitted manuscripts and in manuscript tracking systems would support more accurate editorial review and minimise the risk of misclassification. To facilitate adherence, journals could provide clearer editorial guidance or checklists specifying what registration data must be reported. Editors, rather than peer reviewers, could take responsibility for independently verifying prospective registration and inform reviewers that this has been done. If editors make an exception to the requirement for prospective registration in an ICMJE accepted registry, they should consider explaining their reasons in an editor’s note or another comment on the paper. Journals claiming adherence to the ICMJE recommendations should implement tighter procedures to support the editorial scrutiny needed to prevent the publication of trials not prospectively registered in an ICMJE accepted registry.

Box 1 **Journal requirements for reporting trial registration**

Information journals could request on submission and ensure it is provided in the publication:

Date (day/month/year) trial was first submitted to a trial registry (trial registration date), according to the latest registry entry
Date (day/month/year) the submission was approved by the registry (approval date), according to the latest registry entry
Date (day/month/year) the first participant was enrolled in the trial (enrolment date), according to the latest registry entry
Trial registry name
Trial registration number
Hyperlink to the trial registry entry
Clear indication whether the trial was prospectively registered in an ICMJE accepted registry

On publication, all items should appear both at the end of the abstract and in the main body of the manuscript.
Example of trial registration information to be included in abstracts:
**Trial registration**: Prospectively registered in an ICMJE accepted registry (ClinicalTrials.gov, identifier NCTXXXXXXX [with hyperlink]). First submitted to registry: 25/05/2025. First approved: 10/06/2025. First participant enrolled: 18/06/2025.

Authors should not conflate trial registration with ethical approval, should be aware of which registries the ICJME accepts, and should familiarise themselves with the ICMJE’s definition of a clinical trial. Authors must ensure that trial registration is completed before the enrolment of the first participant using an ICJME approved registry. All relevant registration elements, including exact recruitment dates rather than only randomisation or intervention dates, should be reported in the manuscript. If a trial was retrospectively registered, the reason for the delay should be clearly explained in the submitted manuscript. All research stakeholders, including universities, funders, ethics committees, journals, and registry managers, should cooperate to improve authors’ awareness of these key requirements.

Trial registry managers could work to harmonise terminology, particularly regarding dates, and data elements and structures across registries. The initial date the trial was submitted to the registry should be consistently documented across registries and could be used alongside the date the registration was approved by the registry to promote transparency regarding potential administrative or author related delays. Registries could also provide accessible, clear educational resources to guide users in submitting accurate and complete trial registration information.

### Conclusions

This study suggests that larger sample sizes, corresponding authors from Oceania, more authors, mention of CONSORT, recent submissions (2021-23), and reported funding are associated with reduced odds of non-prospective registration in an ICMJ accepted registry, whereas trials with corresponding authors from Asia had higher odds. Many trials rejected by *The BMJ* for non-prospective registration in an ICMJE accepted registry were later published in high impact journals claiming adherence to the ICMJE recommendations, often without disclosure of registration deficiencies. Strengthened editorial examination, clearer registry standards, and increased author awareness are needed to improve transparency of registration practices and reporting. We suggest actions for journal editors, authors, and registry managers.

**What is already known on this topic**

The International Committee of Medical Journal Editors (ICMJE) mandated that, beginning in July 2005, clinical trials must be prospectively registered (on or before enrolment of the first participant) in an ICMJE accepted registry to be eligible for publication in ICMJE member journals
Accepted registries include the 18 primary registries of the WHO International Clinical Trials Registry Platform as well as ClinicalTrials.gov, the Dutch Trial Register, EudraCT, and the UMIN Clinical Trials Registry.
Trial registration rates have improved over time, but trials not prospectively registered in an ICMJE accepted registry are still being identified

**What this study adds**

Larger sample sizes, corresponding authors from Oceania, more authors, mention of the Consolidated Standards Of Reporting Trials, recent submissions, and reported funding are associated with reduced odds of non-prospective registration in an ICMJE accepted registry
Many trials not prospectively registered in an ICMJE accepted registry continue to be published in high impact journals claiming adherence to the ICMJE recommendations, often without disclosing deficiencies in registration
Journals could require authors to report the exact dates of registry submission and approval, and first participant enrolled; registry name and number; a hyperlink to the registry entry; and indication whether the trial was prospectively registered in an ICMJE accepted registry

## Data Availability

The anonymised dataset used for the analysis is available in Zenodo.^21^ All information has been removed that could identify the manuscript, including the submission IDs, titles, abstracts, authors’ names, registration numbers, and publication identifiers. The codes used for statistical analysis^21^ and text matching data extraction^18^ are available in Zenodo.

## Web extra

Extra material supplied by authors

Supplementary information: File 1: list of ICMJE accepted registries; file 2: criteria used for the classification of drug interventions; file 3: R packages used for the statistical analysis; and file 4: calibration plot for model validation

## Notes

Contributors: DB, EL, SC, and SS conceived the study. DB, VZ, and SS curated the data. DB, MC, and JC carried out the formal analysis. DB, SC, ACA, EH, and SS undertook the investigation. DB and JC developed the methodology. DB and SS oversaw the project administration. DB, MC, JC, and VZ developed the software. DB and JC were responsible for the data visualisation. SS provided the resources. DB wrote the original draft. EL, SC, MC, JC, ACA, VZ, EH, and SS reviewed and edited the manuscript. DB is the guarantor and accepts full responsibility for the work and the integrity of the data. The corresponding author attests that all listed authors meet authorship criteria and that no others meeting the criteria have been omitted.

Transparency: The lead author affirms that the manuscript is an honest, accurate, and transparent account of the study being reported; that no important aspects of the study have been omitted; and that any discrepancies from the study as originally planned (and, if relevant, registered) have been explained.

Dissemination to participants and related patient and public communities: This was a descriptive study with no research participants. The relevant community for dissemination of results is editors and publishers, so we presented the work at the 10th international Congress on Peer Review and Scientific Publication.

Provenance and peer review: Not commissioned; externally peer reviewed.

## References

[1] International Committee of Medical Journal Editors. Clinical Trials Registration. [Cited 9 Apr 2025.] https://www.icmje.org/about-icmje/faqs/clinical-trials-registration/

[2] Lindsley K Fusco N Li T Scholten R Hooft L . Clinical trial registration was associated with lower risk of bias compared with non-registered trials among trials included in systematic reviews. J Clin Epidemiol 2022;145:164-73. 10.1016/j.jclinepi.2022.01.012. 35081449 PMC9875740

[3] De Angelis C Drazen JM Frizelle FA International Committee of Medical Journal Editors . Clinical trial registration: a statement from the International Committee of Medical Journal Editors. Ann Intern Med 2004;141:477-8. 10.7326/0003-4819-141-6-200409210-00109. 15355883

[4] Trinquart L Dunn AG Bourgeois FT . Registration of published randomized trials: a systematic review and meta-analysis. BMC Med 2018;16:173. 10.1186/s12916-018-1168-6. 30322399 PMC6190546

[5] Mongin D Buitrago-Garcia D Capderou S . Prospective registration of trials: where we are, why, and how we could get better. J Clin Epidemiol 2024;176:111586. 10.1016/j.jclinepi.2024.111586. 39481460

[6] Klatte K Sluka C Gloy V . Towards full clinical trial registration and results publication: longitudinal meta-research study in Northwestern and Central Switzerland. BMC Med Res Methodol 2023;23:27. 10.1186/s12874-023-01840-9. 36707766 PMC9880919

[7] Al-Durra M Nolan RP Seto E . Prospective registration and reporting of trial number in randomised clinical trials: global cross sectional study of the adoption of ICMJE and Declaration of Helsinki recommendations. BMJ 2025;389:e080945. 10.1136/bmj-2024-080945. 32291261 PMC7190012

[8] Gopal AD Wallach JD Aminawung JA . Adherence to the International Committee of Medical Journal Editors’ (ICMJE) prospective registration policy and implications for outcome integrity: a cross-sectional analysis of trials published in high-impact specialty society journals. Trials 2018;19:448. 10.1186/s13063-018-2825-y. 30134950 PMC6106722

[9] Loder E Loder S Cook S . Characteristics and publication fate of unregistered and retrospectively registered clinical trials submitted to *The BMJ* over 4 years. BMJ Open 2018;8:e020037. 10.1136/bmjopen-2017-020037. 29453302 PMC5829901

[10] von Elm E Altman DG Egger M Pocock SJ Gøtzsche PC Vandenbroucke JP STROBE Initiative . The Strengthening the Reporting of Observational Studies in Epidemiology (STROBE) statement: guidelines for reporting observational studies. J Clin Epidemiol 2008;61:344-9. 10.1016/j.jclinepi.2007.11.008. 18313558

[11] International Committee of Medical Journal Editors. Clinical Trials. [Cited 23 Jan 2025.] https://www.icmje.org/recommendations/browse/publishing-and-editorial-issues/clinical-trial-registration.html

[12] International Committee of Medical Journal Editors. Primary registries in the WHO registry network. [Cited 20 Nov 2025.] https://www.who.int/tools/clinical-trials-registry-platform/network/primary-registries

[13] Holst M Carlisle BG . Trials that turn from retrospectively registered to prospectively registered: a cohort study of “retroactively prospective” clinical trial registration using history data. Trials 2024;25:189. 10.1186/s13063-024-08029-5. 38486299 PMC10938677

[14] Schulz KF Altman DG Moher D CONSORT Group . CONSORT 2010 statement: updated guidelines for reporting parallel group randomised trials. BMJ 2010;340:c332. 10.1136/bmj.c332. 20332509 PMC2844940

[15] The SPIRIT–CONSORT Group. Extensions of the SPIRIT and CONSORT Statements. [Cited 9 Jun 2025.] https://www.consort-spirit.org/extensions

[16] Gayet-Ageron A Ben Messaoud K Richards M . Gender and geographical bias in the editorial decision-making process of biomedical journals: a case-control study. BMJ Evid Based Med 2025;30:149-62. 10.1136/bmjebm-2024-113083 PMC12171469 39721743

[17] Python Software Foundation. Python 3.12.5: Release Date 6 August 2024. 2024. [Cited 6 Jun 2025.] https://www.python.org/doc/

[18] Zárate V. Text-matching code (trial registration project). [Cited 20 Jun 2025.] https://zenodo.org/records/15703637

[19] International Committee of Medical Journal Editors. ICMJE Ceases List of Journals Claiming to Follow Its Recommendations. [Cited 13 Oct 2025.] https://www.icmje.org/news-and-editorials/updated_recommendations_apr2025.html

[20] Rothman KJ Greenland S Lash TL . Modern Epidemiology. 3rd ed. Lippincott Williams & Wilkins, 2008.

[21] Blanco D, Schroter S, Cortés J. Databases and R code used for the analysis (trial registration project). [Cited 20 Nov 2025.] https://zenodo.org/records/17661977

[22] R Core Team. R: A language and environment for statistical computing. Version 4.5.0. 2024. [Cited 6 Jun 2025.] https://www.R-project.org/

[23] Hunter KE Seidler AL Askie LM . Prospective registration trends, reasons for retrospective registration and mechanisms to increase prospective registration compliance: descriptive analysis and survey. BMJ Open 2018;8:e019983. 10.1136/bmjopen-2017-019983. 29496896 PMC5855169

[24] Gresham G Meinert JL Gresham AG Piantadosi S Meinert CL . Update on the clinical trial landscape: analysis of ClinicalTrials.gov registration data, 2000-2020. Trials 2022;23:858. 10.1186/s13063-022-06569-2. 36203212 PMC9540299

[25] Hoffmann JM Grossmann R Widmann A . Academic clinical trials: Publication of study results on an international registry-We can do better! Front Med (Lausanne) 2022;9:1069933. 10.3389/fmed.2022.1069933. 36507494 PMC9729766

[26] Hopewell S Chan AW Collins GS . CONSORT 2025 statement: updated guideline for reporting randomised trials. BMJ 2025;389:e081123. 10.1136/bmj-2024-081123. 40228833 PMC11995449

[27] Cole GD Nowbar AN Mielewczik M Shun-Shin MJ Francis DP . Frequency of discrepancies in retracted clinical trial reports versus unretracted reports: blinded case-control study. BMJ 2015;351:h4708. 10.1136/bmj.h4708. 26387520 PMC4575810

[28] Health Research Authority. Clinical Trial Registration Audit Report. [Cited 9 Jun 2025.] https://www.hra.nhs.uk/planning-and-improving-research/policies-standards-legislation/research-transparency/registering-research-studies/clinical-trial-registration-audit-report/

[29] Trung LQ Morra ME Truong ND . A systematic review finds underreporting of ethics approval, informed consent, and incentives in clinical trials. J Clin Epidemiol 2017;91:80-6. 10.1016/j.jclinepi.2017.08.007. 28866123

