# Supplementary material for: Analysis of non-prospective trial registration in clinical trials submitted to The BMJ: observational study
Source: BMJ. 2026 Feb 18;392:e086467. doi: 10.1136/bmj-2025-086467 (PMC12914382; doi:10.1136/bmj-2025-086467)

File 1: List of ICMJE-accepted registries

- Australian New Zealand Clinical Trials Registry (ANZCTR)
- Brazilian Clinical Trials Registry (ReBEC)
- Chinese Clinical Trial Registry (ChiCTR)
- Clinical Research Information Service, Republic of Korea (CRiS)
- ClinicalTrials.gov
- Clinical Trials Information System (CTIS)
- Clinical Trials Registry – India (CTRI)
- Cuban Public Registry of Clinical Trials (RPCEC)
- EU Clinical Trials Register (EU-CTR)
- EudraCT (EU Drug Reg. CTR)
- German Clinical Trials Register (DRKS)
- Iranian Registry of Clinical Trials (IRCT)
- ISRCTN registry (ISRCTN)
- International Traditional Medicine Clinical Trial Registry (ITMCTR)
- Japan Registry of Clinical Trials (jRCT)
- Lebanese Clinical Trials Registry (LBCTR)
- Netherlands Trial Register (NTR / Onderzoek met Mensen)
- Pan African Clinical Trial Registry (PACTR)
- Peruvian Clinical Trial Registry (REPEC)
- Sri Lanka Clinical Trials Registry (SLCTR)
- Thai Clinical Trials Registry (TCTR)
- UMN Clinical Trials Registry (UMIN-CTR)

File 2: Criteria used for the classification of pharmacologic interventions.

We classified as pharmacological interventions those that involved the use of drugs or medications to treat or prevent a disease. Non-pharmacological interventions included psychotherapy, exercise, physiotherapy, education, behavioural interventions, and surgical procedures.

The following specific cases were classified as follows:

1. Vaccines, hormone therapies – pharmacological
2. Oxygen, intravenous fluids – pharmacological
3. Nutritional supplements (vitamins, etc.) – pharmacological if used to treat a specific condition, non-pharmacological if used for general wellness
4. Herbal medicine – pharmacological if they involve ingesting or applying a medicinal substance to treat or prevent a condition, non-pharmacological otherwise
5. Homeopathy – pharmacological
6. Trials where a drug or medication was involved but the intervention was procedural or tangential (e.g., timing of antibiotics, nurse vs doctor titrating medication regime, genotyping vs not genotyping for drug metabolism) – non-pharmacological
7. Human tissues/cells/microbes (e.g., faecal microbiota transplantation and convalescent plasma) – non-pharmacological
8. Radiotherapy – non-pharmacological

File 3: *R* packages used for the statistical analysis

We used the following R packages: *compareGroups* for descriptive statistics and group comparisons; *dplyr* for data manipulation and preprocessing, *stats* for model fitting and inference; *car* for regression diagnostics; *effects* and *emmeans* for estimating and visualising marginal effects, *broom* for tidying model outputs; *predtools* for predictive performance evaluation, generating calibration plots to compare predicted with observed probabilities; *flextable* and *officer* to generate tables suitable for Word documents; and *openxlsx* to export results to Excel files.

File 4: Calibration plot for model validation.

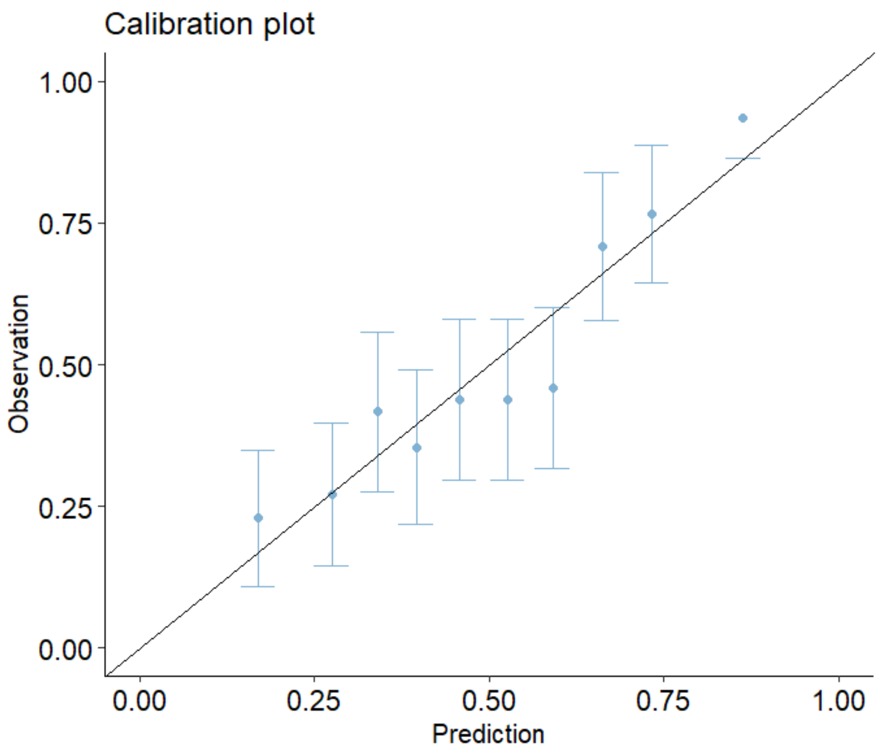

Supplement: Supplementary file 1 — Supplementary information: File 1: list of ICMJE accepted registries; file 2: criteria used for the classification of drug interventions; file 3: R packages used for the statistical analysis; and file 4: calibration plot for model validation [file blad086467.ww.pdf]
